# Integrative transcriptomic analysis of peripheral blood identifies NETosis-associated programs and a stratified adaptive immune suppression pattern in severe acute pancreatitis

**DOI:** 10.1371/journal.pone.0349692

**Published:** 2026-09-25

**Authors:** Yaobin He, Kairui Liu, Rong Chen, Yipei Huang, Youxing Huang

**Affiliations:** Department of Abdominal Surgery, The Second Affiliated Hospital of Guangzhou University of Chinese Medicine, Guangzhou, Guangdong Province, China; Pacific Northwest National Laboratory, UNITED STATES OF AMERICA

## Abstract

**Background:**

Severe acute pancreatitis (SAP) is associated with persistent organ failure and substantial mortality, yet the transcriptomic mechanisms underlying progression from mild acute pancreatitis to severe-spectrum disease remain incompletely defined. Early identification of patients at risk of persistent organ failure remains an important unmet clinical need.

**Methods:**

We performed an integrative transcriptomic analysis of peripheral blood RNA-seq data from GSE194331 (healthy controls [HC], n = 32; mild acute pancreatitis [MAP], n = 57; severe-spectrum AP group [SAP group], n = 30, comprising MSAP n = 20 and severe AP n = 10; sampled within 24 hours of admission). A dual-comparison framework was established using SAP vs HC and SAP vs MAP to distinguish broad AP-associated changes from organ failure-associated features. Gene set enrichment analysis (GSEA), ssGSEA-based immune signature analysis, triple-algorithm machine learning (LASSO, random forest, and SVM-RFE), and weighted gene co-expression network analysis (WGCNA) were integrated to identify candidate genes and characterize associated biological programs. Cross-species single-cell RNA-seq data from rat ileal tissue (GSE244963) were used only as supportive cell-type context for candidate genes.

**Results:**

GSEA identified enrichment of NETosis, complement/coagulation, oxidative phosphorylation, and multiple inflammatory cell death-related programs in the SAP group. ssGSEA revealed reduced adaptive immune-associated signatures, with CD8 + T-cell and NK-cell signatures already reduced in MAP and CD4 + T-cell and Treg-associated signatures showing additional reduction in SAP relative to MAP. Triple-algorithm machine learning identified three core candidate genes with discovery-cohort AUC values: MRPL51 (ML-A, SAP vs HC, AUC = 0.951), C1QA (ML-B, SAP vs MAP, AUC = 0.754), and DACT1 (ML-B, SAP vs MAP, AUC = 0.758). Repeated stratified 5-fold cross-validation supported internal stability of the single-gene estimates, but no independent external validation cohort was available. A neutrophil-associated NETosis gene triad (OLFM4, LTF, and CEACAM6) was additionally co-selected by LASSO and random forest in ML-B. Rat ileal scRNA-seq provided supportive, cross-species cell-type context rather than definitive validation.

**Conclusion:**

Integrative transcriptomic analysis of peripheral blood identifies candidate transcriptomic markers and suggests neutrophil-associated, metabolic, and adaptive immune programs associated with AP severity progression. The stratified adaptive immune signature reduction pattern and the NETosis-associated gene triad may represent discovery-level transcriptomic features of progression from mild AP to the severe-spectrum AP group. Independent validation in prospective human cohorts is required.

## 1. Introduction

Acute pancreatitis (AP) is one of the most common gastrointestinal emergencies worldwide, with an estimated global incidence of 34 per 100,000 person-years and a rising trend over recent decades [1]. According to the 2012 Revised Atlanta Classification, AP is classified as mild acute pancreatitis (MAP), moderately severe acute pancreatitis (MSAP), and severe acute pancreatitis (SAP) based on the presence and duration of organ failure [2]. While the majority of patients experience a self-limiting course, severe acute pancreatitis is associated with persistent organ failure, systemic inflammatory dysregulation, and a reported mortality of approximately 20–40% [3]. The early phase of AP is clinically critical, and persistence of organ failure beyond 48 hours is a key indicator of poor prognosis and severe disease [4]. Yet reliable early predictors of this transition remain elusive in routine clinical practice.

The progression of SAP is associated with dysregulated innate immunity, excessive systemic inflammation, and subsequent immune imbalance. Among these processes, neutrophil extracellular trap formation (NETosis) has been increasingly implicated as an important contributor to pancreatic injury, complement activation, and microvascular dysfunction [5]. Multiple inflammatory cell death-related programs, including pyroptosis and ferroptosis, may also contribute to tissue damage amplification, and their concurrent activation in SAP fits with coordinated inflammatory cell-death states described in other inflammatory contexts [6]. In parallel, SAP may be accompanied by suppression of adaptive immune responses, a pattern aligned with the compensatory anti-inflammatory response syndrome (CARS) framework originally proposed in sepsis and critical illness [7]. The complement system has been implicated in AP-associated systemic inflammation, with substantial crosstalk between complement activation and coagulation pathways during organ injury [8]. However, the transcriptomic architecture underlying these interconnected processes during progression from MAP to SAP remains incompletely defined.

Peripheral blood transcriptomics offers a practical and minimally invasive approach to dissect systemic immune states in AP. Bulk RNA sequencing of peripheral blood captured within 24 hours of admission reflects the early systemic immune response and provides a tractable window for biomarker discovery. Computational deconvolution methods such as single-sample gene set enrichment analysis (ssGSEA) enable estimation of relative immune cell-associated signature enrichment scores from bulk transcriptomic data without requiring cell sorting [9]. Machine learning-based feature selection has been increasingly applied to omics data for biomarker discovery and patient stratification, supporting the use of complementary algorithms to improve candidate prioritization [10]. Weighted gene co-expression network analysis (WGCNA) further allows candidate genes to be contextualized within co-expression modules that reflect coordinated biological programs rather than individual gene effects [11].

Although transcriptomic studies have begun to profile severity-associated molecular changes in acute pancreatitis, including bulk and single-cell analyses of severe disease, the organ failure-focused transcriptomic architecture underlying progression from mild AP to severe-spectrum disease remains incompletely defined [12,13]. Furthermore, machine learning approaches applied to AP transcriptomics have rarely reported full feature-selection thresholds, random seeds, and cross-validation settings, raising concerns about reproducibility. The potential for a stratified pattern of immune signature reduction in which different adaptive immune-associated signatures change at different stages of disease progression has also not been examined systematically at the transcriptomic level.

In this study, we performed an integrative transcriptomic analysis of peripheral blood RNA-seq data from GSE194331, including healthy controls (HC) and AP patients across different severity strata sampled within 24 hours of admission. We adopted a dual-comparison framework (SAP vs HC and SAP vs MAP) to distinguish broad AP-associated changes from severe-spectrum features related to organ failure. By integrating GSEA, ssGSEA-based immune signature analysis, multi-algorithm machine learning, and WGCNA, we aimed to identify biologically interpretable pathways and candidate biomarkers associated with AP severity progression. In addition, cross-species single-cell RNA-seq data from rat ileal tissue were used to provide supportive cell-type context for selected candidate genes.

## 2. Materials and methods

### 2.1. Data source and study cohort

Publicly available transcriptomic data were obtained from the Gene Expression Omnibus (GEO) database (accession number GSE194331). This dataset comprises peripheral blood RNA sequencing (RNA-seq) data from 119 participants collected within 24 hours of hospital admission, including 87 patients with acute pancreatitis (AP) and 32 healthy controls (HC). AP patients were classified according to the 2012 Revised Atlanta Classification [2]. For downstream binary severity analyses, patients with moderately severe AP (MSAP, n = 20; transient organ failure) and severe AP (n = 10; persistent organ failure) were combined into a single severe-spectrum AP group (hereafter referred to as the SAP group, n = 30), while patients without organ failure were defined as the MAP group (n = 57). This grouping was selected to increase statistical power for organ-failure-associated transcriptomic analyses and was evaluated by sensitivity analyses comparing MSAP with true SAP. The original dataset was published by Nesvaderani et al [12]. No additional ethical approval was required as all data were retrieved from publicly accessible repositories. A second public dataset, GSE244963, comprising scRNA-seq data from rat ileal tissue (SAP n = 3, normal control [NC] n = 3), was used for supportive cross-species cell-type contextualization only. Cohort characteristics and dataset details are summarized in Table 1.

**Table 1:** Study cohort characteristics and dataset overview.

Characteristic	HC (n = 32)	MAP (n = 57)	SAP (n = 30)
**Dataset**			
GEO accession	GSE194331	GSE194331	GSE194331
Sample type	Peripheral blood RNA-seq	Peripheral blood RNA-seq	Peripheral blood RNA-seq
Collection timepoint	Within 24 h of admission	Within 24 h of admission	Within 24 h of admission
Reference	Nesvaderani et al., J Am Coll Surg 2022	Nesvaderani et al., J Am Coll Surg 2022	Nesvaderani et al., J Am Coll Surg 2022
**Clinical classification**			
Atlanta criteria	Healthy volunteer	Mild AP, no organ failure	Organ failure present
Subgroup	—	MAP group	MSAP (n = 20) + SAP (n = 10)
Severe-spectrum grouping	—	MAP group	SAP group (MSAP+SAP combined)
**Transcriptomics**			
Total samples	32	57	30
DEGs vs HC (|log2FC| > 1, FDR < 0.05)	—	366	2,002
DEGs SAP vs MAP (|log2FC| > 1, FDR < 0.05)	—	—	127

HC, healthy control; MAP, mild acute pancreatitis; SAP, severe-spectrum acute pancreatitis. For downstream analyses, patients with moderately severe AP (MSAP, n = 20) and severe AP (n = 10) were combined into a single severe-spectrum group (SAP group, n = 30). DEG, differentially expressed gene; FDR, false discovery rate. Differential expression analysis performed using limma-voom with thresholds |log2FC| > 1 and FDR < 0.05.

To assess whether combining MSAP and severe AP distorted the severe-spectrum analysis, we performed post hoc sensitivity analyses comparing MSAP (n = 20) with true severe AP (n = 10). These analyses included limma-voom differential expression, PCA/clustering using the top 2,000 median-absolute-deviation genes, Wilcoxon tests for candidate genes, and ssGSEA immune-signature comparisons. Results were interpreted cautiously because the true SAP subgroup was small and absence of significance does not prove equivalence.

### 2.2. Data preprocessing and normalization

Raw count matrices from GSE194331 were imported into R (version 4.3). Normalization was performed using the trimmed mean of M-values (TMM) method implemented in the edgeR package, followed by conversion to log2-counts per million (log2-CPM) for downstream visualization and machine learning analyses. Ensembl gene IDs were mapped to HGNC gene symbols using a custom annotation file (48,698 entries) derived from Ensembl release 109.

### 2.3. Differential expression analysis

Differential expression analysis was performed using the limma-voom pipeline. Three pairwise comparisons were conducted: SAP vs HC, MAP vs HC, and SAP vs MAP. Differentially expressed genes (DEGs) were defined by an absolute log2 fold change (|log2FC|) > 1 and a Benjamini-Hochberg-adjusted false discovery rate (FDR) < 0.05. The SAP vs HC DEG list (n = 2,002) served as input for the ML-A framework, and the SAP vs MAP DEG list (n = 127) served as input for the ML-B framework.

### 2.4. Gene set enrichment analysis

Gene Set Enrichment Analysis (GSEA) was conducted using the GSEApy package (version 1.1.3) in Python. Genes were ranked by moderated t-statistics obtained from the limma-voom model for each comparison. Pathway analysis incorporated curated gene sets from MSigDB collections (Hallmark, KEGG 2021 Human, and Reactome). We pre-specified 15 AP-relevant pathways because they represent inflammatory, neutrophil, complement/coagulation, metabolic, cell-death, and adaptive-immune processes implicated in AP severity. Custom literature-derived gene sets related to inflammasome activation, pyroptosis, NETosis, PANoptosis, cuproptosis, necroptosis, ferroptosis, sepsis/organ failure, and mitochondrial dysfunction were additionally tested. Full GMT-compatible membership lists are provided in Supplementary Table S6 in S1 File.

### 2.5. Immune signature enrichment scoring

Relative immune cell-associated enrichment scores in peripheral blood transcriptomes were estimated using single-sample GSEA (ssGSEA), implemented in the GSVA package in R [9]. A curated panel of nine 10-gene signatures was applied: neutrophils, monocytes, M1 macrophages, NK cells, CD4 + naive T cells, regulatory T cells (Treg), CD8 + effector T cells, CD8 + exhausted T cells, and ferroptosis-defense genes. Complete signature membership is provided in Supplementary Table S7 in S1 File. Between-group differences across HC, MAP, and SAP were assessed using non-parametric tests with Benjamini-Hochberg FDR correction. Because these scores are transcriptomic enrichment estimates rather than direct cell counts, we refer to them as immune signature enrichment rather than immune infiltration.

### 2.6. Dual-framework machine learning for biomarker identification

A dual-framework machine learning strategy was implemented: ML-A used the 2,002 DEGs from SAP vs HC; ML-B used the 127 DEGs from SAP vs MAP. Within each framework, three independent feature selection algorithms were applied: (1) Least Absolute Shrinkage and Selection Operator (LASSO) logistic regression with 5-fold cross-validation (`LassoCV`, maximum iterations = 10,000, random_state = 42); (2) Random Forest (RF) with 500 trees, random_state = 42, and a fixed top-50 Gini-importance cutoff; and (3) Support Vector Machine with Recursive Feature Elimination (SVM-RFE) using a linear kernel, C = 1, step = 0.1, and feature number chosen by maximum 5-fold cross-validation accuracy [10]. Only genes consistently selected by all three algorithms within the same framework were retained as consensus candidates. Single-gene diagnostic performance was reported as discovery-cohort ROC AUC using raw expression values for consistency with the primary ROC analysis, and internal robustness was further assessed using logistic-regression LOOCV and repeated stratified 5-fold cross-validation (5 folds x 20 repeats). All machine-learning analyses were implemented in Python using scikit-learn (version 1.7.2), with PYTHONHASHSEED = 42 and numpy random seed = 42.

### 2.7. Weighted gene co-expression network analysis

Weighted gene co-expression network analysis (WGCNA) was performed using the supplied Python script `step6_wgcna.py`, following the workflow of the WGCNA R package [11]. The top 5,000 most variable genes, selected by median absolute deviation (MAD), were used for network construction. A soft-thresholding power of beta = 9 was selected as a compromise between scale-free topology fit (R2 = 0.806) and network connectivity in this small exploratory cohort (S3 Fig). Dynamic tree cutting identified six co-expression modules. Module eigengenes were correlated with an ordinal severity variable coded from HC to MAP to SAP using Pearson correlation. Full module gene membership is provided in Supplementary Table S8 in S1 File.

### 2.8. Single-cell RNA sequencing contextualization

To provide supportive cross-species, single-cell-level context for candidate gene expression, we analyzed scRNA-seq data from rat ileal tissue (GSE244963). A custom Python loader was implemented to handle non-standard barcode formatting (see Supplementary Methods SM 2.2). Data processing was performed using scanpy (version 1.11.5). Quality control filters excluded cells with fewer than 200 detected genes, more than 6,000 detected genes, or mitochondrial gene fraction exceeding 20%. After quality control, 80,256 cells were retained. Leiden clustering (resolution = 0.5) was applied to identify cell clusters. Cell type annotation was performed based on canonical marker genes and cluster-level expression patterns. Because this dataset is rat ileal tissue rather than human peripheral blood, it was interpreted only as supportive cell-type context and not as independent validation of human blood biomarkers.

### 2.9. Statistical analysis

All statistical analyses were performed in R (version 4.3) or Python (version 3.10) unless otherwise specified. Unless otherwise specified, multiple testing correction was performed using the Benjamini-Hochberg procedure, and adjusted p values (FDR) < 0.05 were considered statistically significant. Data visualization was performed using ggplot2, ComplexHeatmap, EnhancedVolcano, pROC, and pheatmap in R.

## 3. Results

### 3.1. Peripheral blood transcriptomes show extensive differential expression across AP severity groups

**Fig 1:**
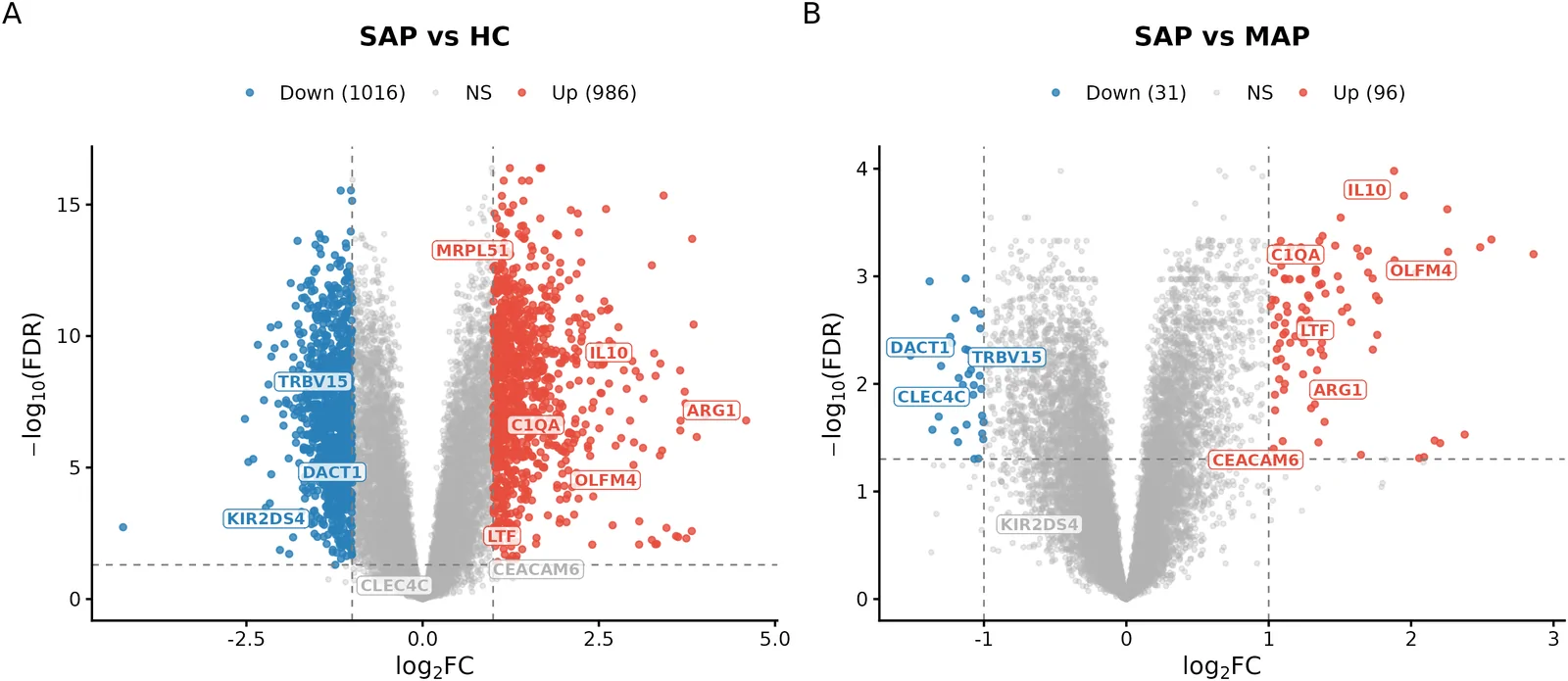
Differential expression analysis of peripheral blood transcriptomes across AP severity groups. Volcano plots illustrating differentially expressed genes (DEGs) identified by limma-voom analysis in (A) SAP vs HC and (B) SAP vs MAP comparisons. The x-axis represents log2 fold change (log2FC) and the y-axis represents −log10(FDR-adjusted p-value). Genes meeting the significance threshold (|log2FC| > 1 and FDR < 0.05) are highlighted: red, upregulated; blue, downregulated; grey, not significant. The total number of significant DEGs, upregulated genes, and downregulated genes are annotated for each comparison. Selected genes of interest are labeled. HC, healthy control; MAP, mild acute pancreatitis; SAP, severe-spectrum acute pancreatitis (includes moderately severe AP and severe AP combined).

To identify transcriptomic alterations associated with AP severity, we performed limma-voom differential expression analysis in the GSE194331 cohort (HC = 32, MAP = 57, SAP = 30). Using |log2FC| > 1 and FDR < 0.05 as thresholds, we identified 2,002 DEGs in SAP vs HC (986 upregulated, 1,016 downregulated) and 127 DEGs in SAP vs MAP (96 upregulated, 31 downregulated) (Fig 1, Table 2). The markedly smaller DEG set in SAP vs MAP than in SAP vs HC suggests that progression from MAP to SAP is characterized by a more restricted but biologically focused transcriptional shift. These two comparisons were therefore used as dual analytical frameworks to distinguish broad AP-associated changes from organ failure-related features.

**Table 2:** Differential expression analysis summary (limma-voom).

Comparison	Total DEGs	Up-regulated	Down-regulated	Application
**SAP vs HC**	2,002	986	1,016	ML-A framework input (pan-AP severity markers)
**MAP vs HC**	366	310	56	Background reference
**SAP vs MAP**	127	96	31	ML-B framework input (organ failure-specific markers)

DEG, differentially expressed gene; ML-A, machine learning framework A (SAP vs HC); ML-B, machine learning framework B (SAP vs MAP). Thresholds: |log2FC| > 1 and FDR < 0.05. Full gene lists are available in S2 File (worksheets DEG_sig_SAP_vs_HC and DEG_sig_SAP_vs_MAP).

Post hoc sensitivity analyses comparing MSAP (n = 20) with true severe AP (n = 10) supported the use of a combined severe-spectrum AP group for the primary organ-failure analysis, while also revealing a graded neutrophil signal within the severe spectrum. No genes reached FDR < 0.05 in the transcriptome-wide MSAP-vs-true-SAP limma-voom analysis, and PCA/clustering showed substantial overlap. Among candidate genes, OLFM4 was higher in true SAP than in MSAP (FDR = 0.008), and LTF showed the same direction at nominal significance (p = 0.019; FDR = 0.057). In contrast, the adaptive-immune ssGSEA signatures selected for sensitivity testing (CD4_Treg, CD8_T_effector, and NK_cells) did not differ significantly. We therefore used the combined severe-spectrum group as a power-preserving analytic choice, while interpreting neutrophil/NETosis-associated markers as graded across the severe spectrum rather than uniform between MSAP and true SAP.

### 3.2. GSEA identifies innate immune activation, metabolic rewiring, and adaptive immune suppression associated with SAP progression

**Fig 2:**
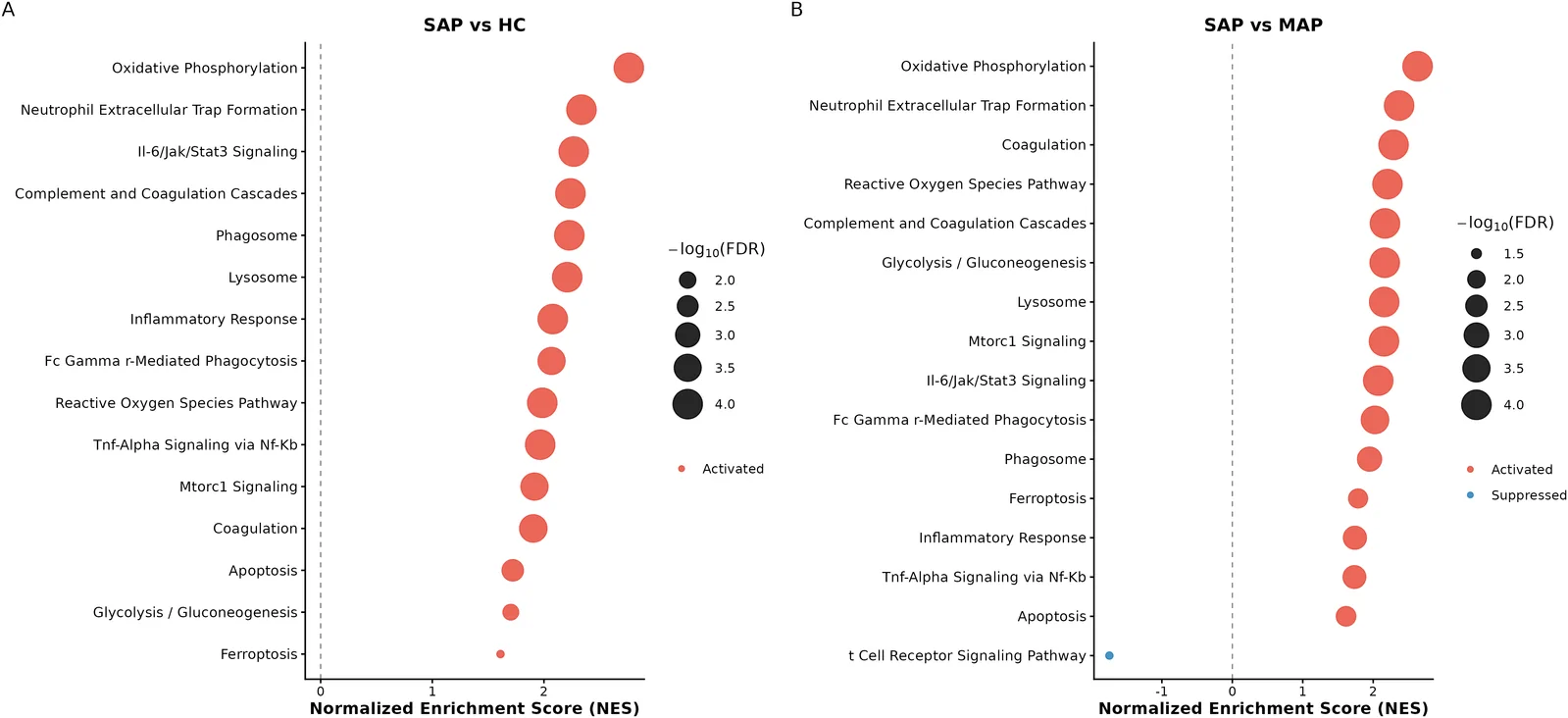
Gene set enrichment analysis reveals innate immune activation and adaptive immune suppression associated with SAP progression. Bubble plots showing GSEA results for 15 pre-specified AP-relevant pathways in (A) SAP vs HC and (B) SAP vs MAP comparisons. Pathways were selected a priori to represent inflammatory, neutrophil, complement/coagulation, metabolic, cell-death, and adaptive-immune processes implicated in AP severity. Bubble size represents the absolute normalized enrichment score (|NES|); bubble color indicates the direction of enrichment (red, activated; blue, suppressed). Only pathways with FDR-adjusted q-value < 0.05 are shown. Pathways were derived from curated Hallmark, KEGG, and Reactome gene set collections. Custom literature-derived gene sets were additionally tested, and complete membership is reported in the Supporting information. NES, normalized enrichment score; FDR, false discovery rate.

Gene set enrichment analysis revealed broad activation of inflammatory and metabolic pathways in SAP. In SAP vs HC, oxidative phosphorylation (NES=+2.76), NETosis (NES=+2.34), IL-6/JAK/STAT3 signaling (NES=+2.27), complement and coagulation cascades (NES=+2.24), phagosome, lysosome, inflammatory response, reactive oxygen species, TNF-α/NF-κB signaling, mTORC1 signaling, apoptosis, glycolysis, and ferroptosis were significantly enriched (Fig 2; Supplementary Table S1 in S1 File). In SAP vs MAP, oxidative phosphorylation (NES=+2.63), complement/coagulation (NES=+2.29), reactive oxygen species (NES=+2.20), glycolysis (NES=+2.16), and ferroptosis (NES=+2.01) remained significantly enriched, while T-cell receptor signaling was the only significantly suppressed pathway (NES < 0), indicating that adaptive immune suppression became more prominent at the organ failure stage.

Using curated custom gene sets, we further observed significant enrichment of inflammasome- (NES=+2.06 SAP vs HC; NES=+1.97 SAP vs MAP), pyroptosis- (NES=+1.99; NES=+1.64), and PANoptosis-related signatures in both comparisons, whereas cuproptosis and necroptosis were not significantly enriched (FDR ≥ 0.05 in both comparisons) (S1 Fig; Supplementary Table S1 in S1 File). Notably, in the custom NETosis gene set, enrichment was stronger in SAP vs MAP (NES=+1.98) than in SAP vs HC (NES=+1.81), supporting a continued association of neutrophil-driven inflammatory programs with progression from MAP to SAP.

### 3.3. Immune signature enrichment analysis suggests a stratified pattern of innate activation and adaptive immune reduction

**Fig 3:**
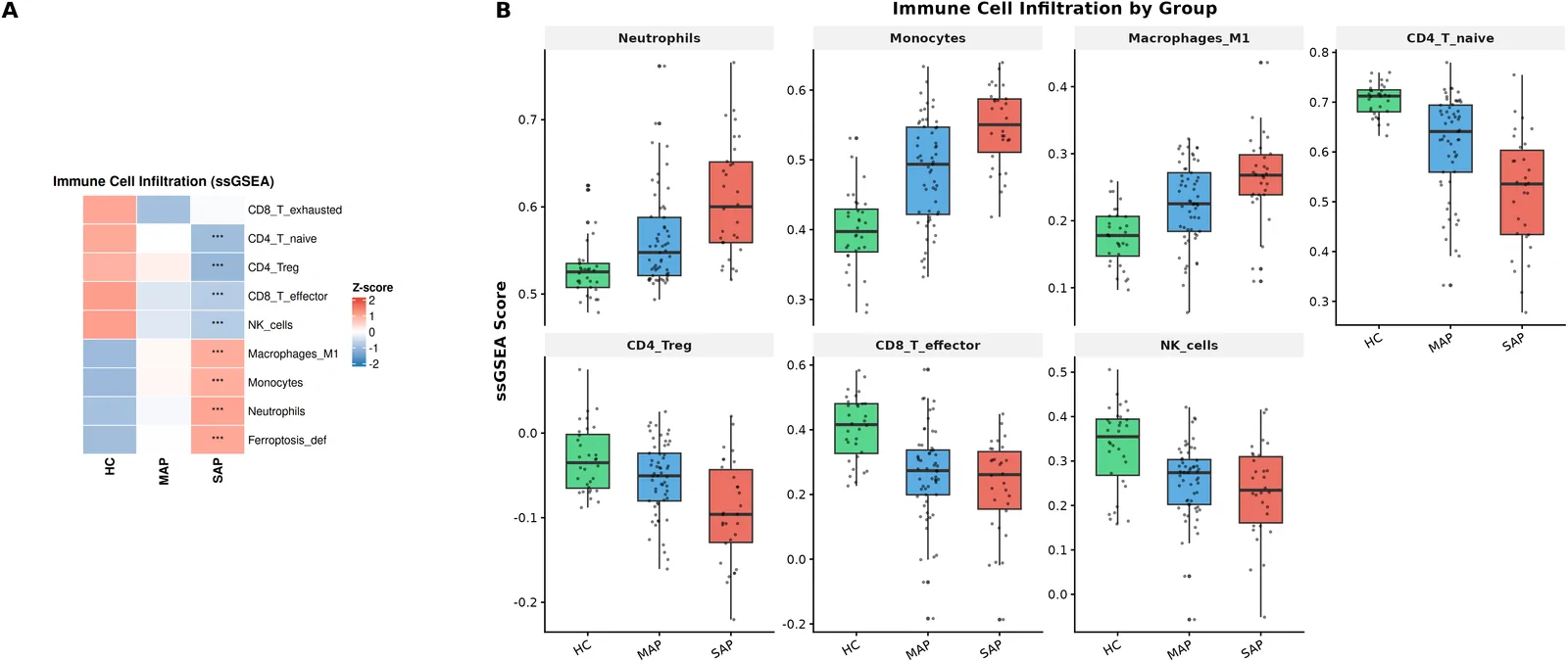
ssGSEA-based immune signature analysis reveals a stratified pattern of innate activation and adaptive immune reduction. (A) Heatmap showing ssGSEA enrichment scores for nine curated 10-gene signatures across all 119 peripheral blood samples, ordered by group (HC, MAP, SAP). Color scale represents row-normalized enrichment scores (red, high; blue, low). (B-H) Boxplots showing pairwise comparisons of ssGSEA scores for selected immune-associated signatures across HC, MAP, and SAP groups. Statistical significance was assessed using non-parametric tests with Benjamini-Hochberg FDR correction. *FDR < 0.05; **FDR < 0.01; ***FDR < 0.001; ns, not significant (FDR >= 0.05). HC, healthy control; MAP, mild acute pancreatitis; SAP, severe-spectrum AP group.

ssGSEA-based immune signature analysis showed progressive remodeling of immune signatures across HC, MAP, and SAP groups (Fig 3; Supplementary Table S2 in S1 File). Innate immune-associated signatures—including neutrophils, monocytes, and M1 macrophages—showed increased enrichment scores with disease severity, consistent with GSEA enrichment of NETosis, complement/coagulation, and inflammatory pathways. In contrast, adaptive immune-associated signatures were generally reduced.

CD8 + T-cell effector and NK-cell signatures were already markedly decreased in MAP relative to HC and did not differ significantly between SAP and MAP (FDR >= 0.05), suggesting these reductions occur as a pan-AP phenomenon at an early disease stage. By contrast, CD4 + T-cell and Treg-associated signatures showed additional reduction in SAP relative to MAP (both FDR < 0.01), indicating an additional layer of adaptive immune signature reduction associated with organ failure. This pattern was consistent with the selective downregulation of T-cell receptor signaling observed only in SAP vs MAP by GSEA, supporting a stratified reduction of adaptive immune signatures.

### 3.4 Dual-framework machine learning identifies three core candidate biomarkers

**Fig 4:**
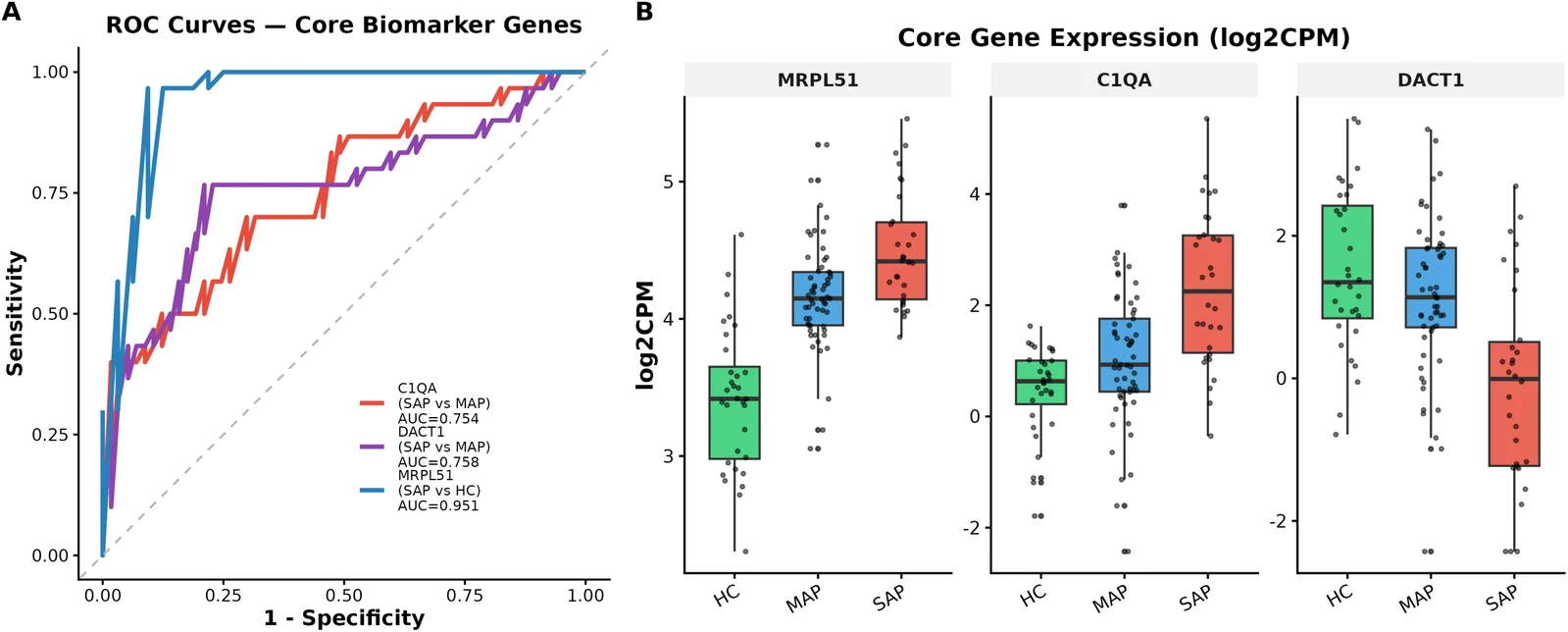
Dual-framework machine learning identifies three core candidate biomarkers. Receiver operating characteristic (ROC) curves for core candidate genes evaluated within the discovery cohort using raw expression values: MRPL51 (ML-A, SAP vs HC, AUC = 0.951), C1QA (ML-B, SAP vs MAP, AUC = 0.754), and DACT1 (ML-B, SAP vs MAP, AUC = 0.758).

To prioritize robust candidate genes, we established two machine-learning frameworks: ML-A based on the 2,002 DEGs from SAP vs HC, and ML-B based on the 127 DEGs from SAP vs MAP. In ML-A, LASSO selected 45 genes, RF retained the top 50 genes by feature importance, SVM-RFE selected 7 genes, and the triple intersection yielded MRPL51. In ML-B, LASSO selected 21 genes, RF retained the top 50 genes, SVM-RFE selected 3 genes, and the triple intersection yielded DACT1 and C1QA (Fig 4).

Three core candidate genes were identified (Table 3). MRPL51, selected from ML-A, was upregulated in SAP vs HC (log2FC=+1.09) and showed the highest discovery-cohort raw-expression ROC performance (AUC = 0.951), in line with strong enrichment of oxidative phosphorylation-related programs. Two ML-B-derived genes, C1QA (log2FC=+1.38, AUC = 0.754) and DACT1 (log2FC = −1.30, AUC = 0.758), differentiated SAP from MAP. Internal robustness checks gave similar but slightly lower logistic-regression estimates: MRPL51 repeated 5-fold AUC = 0.946 + /-0.004, C1QA AUC = 0.746 + /-0.005, and DACT1 AUC = 0.747 + /-0.006 (S5 Fig). These values should be interpreted as discovery-cohort estimates rather than externally validated diagnostic performance. C1QA was linked to macrophage-associated inflammatory activation, whereas reduced DACT1 expression suggested possible Wnt-related dysregulation during SAP progression. HPA annotation indicated that C1QA encodes a secreted protein detectable in plasma, supporting its potential as a circulating biomarker candidate, while DACT1 received the highest HPA reliability classification (Enhanced) (Supplementary Table S3 in S1 File).

**Table 3:** Core machine learning candidate biomarkers.

Gene	Framework	Comparison	log2FC	FDR	Discovery-cohort AUC	WGCNA Module	HPA Reliability
**MRPL51**	ML-A	SAP vs HC	+1.09	<0.001	0.951	— †	Approved
**C1QA**	ML-B	SAP vs MAP	+1.38	<0.001	**0.754**	Red (r=+0.487)	Supported
**DACT1**	ML-B	SAP vs MAP	−1.30	0.007	**0.758**	Brown	Enhanced

AUC, area under the receiver operating characteristic curve evaluated within the discovery cohort using raw expression values; WGCNA, weighted gene co-expression network analysis; HPA, Human Protein Atlas reliability classification. †MRPL51 was excluded from WGCNA high-variance gene filtering due to low median absolute deviation, consistent with its housekeeping gene characteristics; its discovery-cohort single-gene AUC was 0.951. Internal logistic-regression repeated 5-fold cross-validation gave MRPL51 AUC = 0.946 + /-0.004.

In addition, ML-B identified a neutrophil-associated gene triad: OLFM4 (log2FC=+2.26), LTF (log2FC=+1.52), and CEACAM6 (log2FC=+1.10), all upregulated in SAP vs MAP and co-selected by LASSO and random forest (2 of 3 ML-B algorithms) (S4 Fig). Together with GSEA enrichment of NETosis-related programs and progressive neutrophil enrichment scores by ssGSEA, these results associate neutrophil-related inflammatory programs with organ failure progression.

### 3.5 WGCNA co-expression network analysis links candidate genes to severity-associated modules

**Fig 5:**
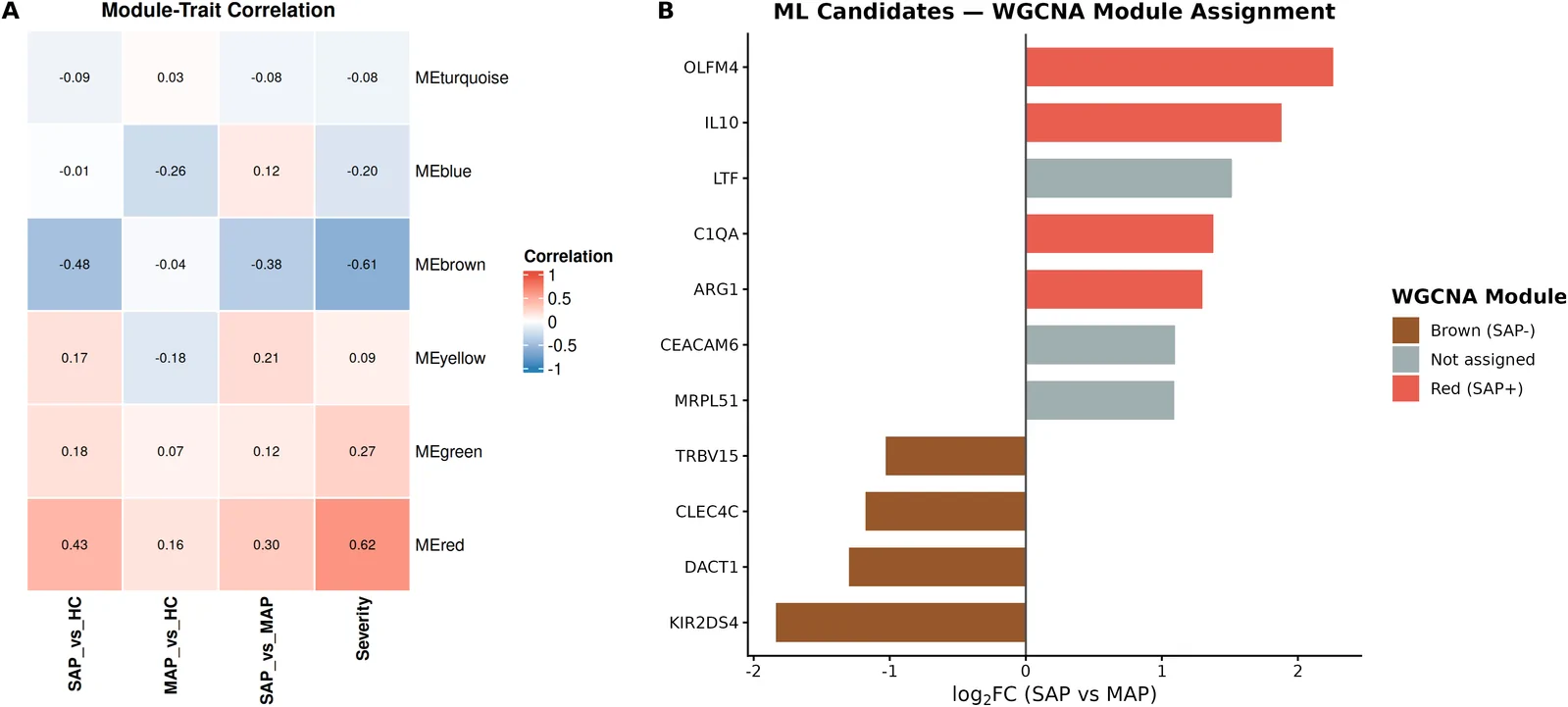
WGCNA co-expression network analysis contextualizes candidate genes within severity-associated modules. (A) Module-trait correlation heatmap showing Pearson correlation coefficients between module eigengenes and trait variables (binary SAP-group membership and an ordinal severity variable coded from HC to MAP to SAP). Color scale represents correlation direction and magnitude (red, positive; blue, negative); values in each cell shows correlation coefficient (upper) and FDR-adjusted p-value (lower). The red module (module 6) showed the strongest positive association with severe-spectrum status and ordinal severity, whereas the brown module (module 3) showed the strongest negative association. (B) Bar chart illustrating the module assignments of machine learning candidate genes, with intra-modular connectivity (Pearson r to module eigengene) shown for genes within the red and brown modules. WGCNA, weighted gene co-expression network analysis.

WGCNA using the top 5,000 highly variable genes identified six co-expression modules (Fig 5; Supplementary Table S4 in S1 File). The red module showed positive correlations with SAP status (r=+0.428) and disease severity (r=+0.623), and included C1QA (intra-modular r=+0.487), OLFM4, IL10, and ARG1, indicating association with innate immune activation and tissue injury-related programs. The brown module was negatively correlated with SAP status (r = −0.481) and severity (r = −0.607), and contained DACT1, CLEC4C, TRBV15, and KIR2DS4, suggesting association with adaptive immune suppression. MRPL51 was not assigned to a severity-associated module, consistent with its broad housekeeping-like expression profile; its high SAP-vs-HC AUC should therefore be interpreted as a discovery-cohort marker of broad AP-associated transcriptional/metabolic differences rather than module-supported organ-failure specificity.

### 3.6 Cross-species scRNA-seq analysis provides supportive cell-type context for candidate genes

**Fig 6:**
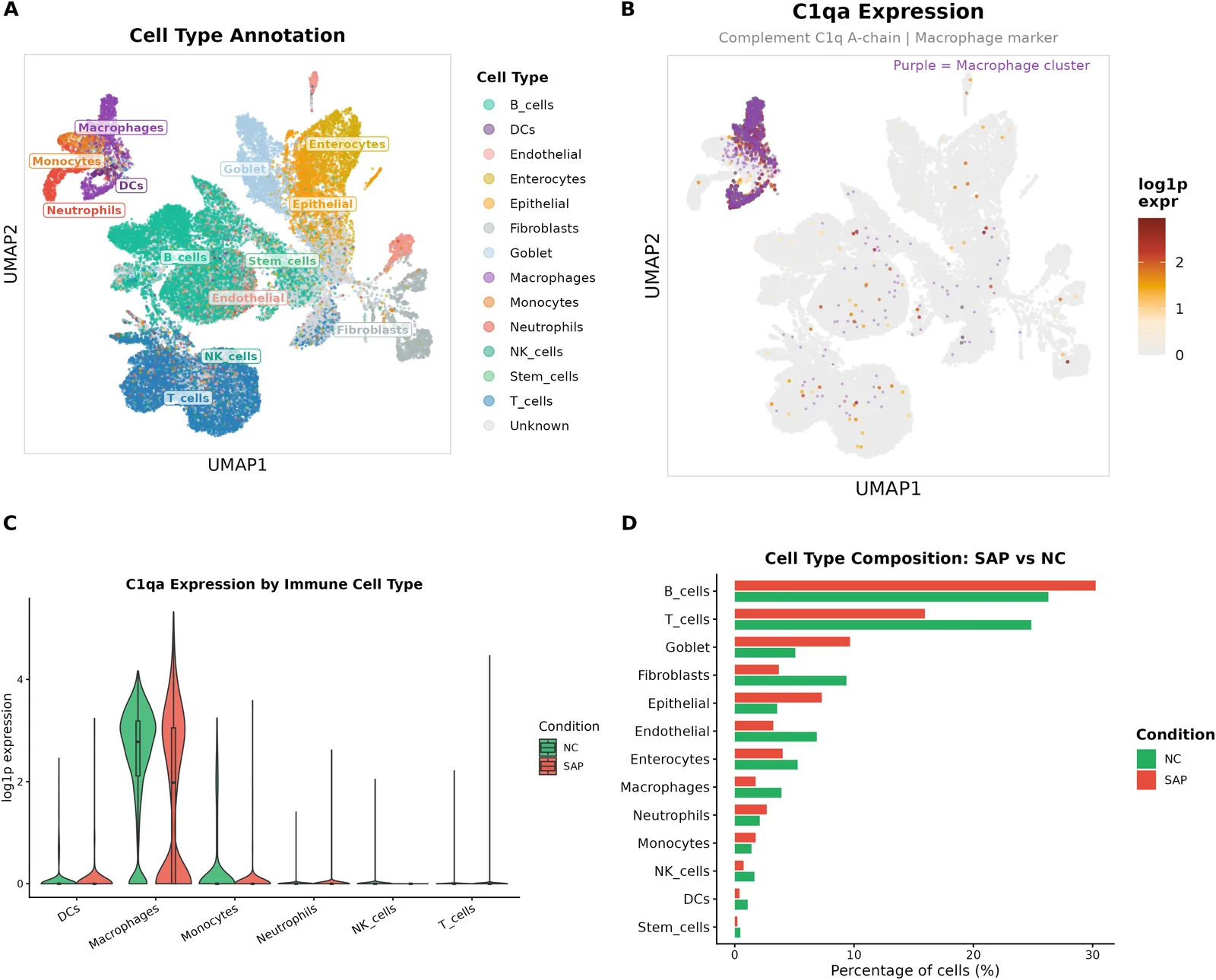
Cross-species single-cell RNA-seq analysis provides supportive cross-species cell-type context for candidate gene expression. Single-cell RNA sequencing data from rat ileal tissue (GSE244963; SAP n = 3, NC n = 3; 80,256 cells post quality control) were analyzed using scanpy (v1.11.5) to provide supportive cross-species cell-type context. (A) UMAP plot showing Leiden-based cell clusters, colored by annotated cell type. (B) UMAP feature plot showing the expression of C1qa across all cells, with color intensity proportional to normalized expression level. (C) Violin plots showing the expression distribution of selected candidate genes (C1qa, Ltf, Arg1, Olfm4, Mrpl51, Dact1) across major cell type clusters in SAP and NC conditions. (D) Bar chart showing cell type composition (proportion of total cells) in SAP versus NC samples. This rat ileal dataset should not be interpreted as independent validation of human peripheral-blood biomarkers. NC, normal control; UMAP, uniform manifold approximation and projection.

We analyzed rat ileal scRNA-seq data (GSE244963; SAP n = 3, NC n = 3), retaining 80,256 cells after quality control (Fig 6; S2 Fig; Supplementary Table S5 in S1 File). Seven of eight candidate genes were detectable. C1qa showed the most prominent macrophage-enriched expression pattern, providing supportive cell-type context consistent with ssGSEA findings. Ltf was enriched in neutrophil-associated regions, supporting the neutrophil origin of the NETosis triad. Arg1 was preferentially expressed in myeloid cells, consistent with its role in myeloid immune suppression. Mrpl51 and Dact1 displayed diffuse low-level expression consistent with multi-tissue expression patterns. Because this dataset was derived from rat ileum rather than human peripheral blood, these findings provide supportive cross-species, tissue-level context but should not be interpreted as definitive validation of the human peripheral-blood biomarkers.

## 4. Discussion

### 4.1. Overview of principal findings

In this study, we performed an integrative transcriptomic analysis of peripheral blood RNA-seq data from a well-characterized acute pancreatitis cohort to characterize discovery-level molecular patterns associated with progression from mild AP to the severe-spectrum AP group. By adopting a dual-comparison framework and integrating GSEA, ssGSEA immune signature analysis, machine learning, and WGCNA, we identified three core candidate genes (MRPL51, C1QA, and DACT1), a neutrophil-associated NETosis triad (OLFM4, LTF, and CEACAM6) co-selected by two ML-B algorithms, and a stratified pattern of adaptive immune signature reduction. Together, the data indicate that severe-spectrum progression is accompanied by neutrophil/NETosis activation, oxidative-metabolic remodeling, and stage-specific reduction of adaptive immune signatures. These observations are internally consistent in the discovery cohort and require independent validation before clinical translation.

### 4.2. NETosis programs and organ-failure progression

One of the clearest severity-related signals in our analysis was the enrichment of NETosis-related programs. In the custom NETosis gene set, enrichment was stronger in SAP vs MAP (NES=+1.98) than in SAP vs HC (NES=+1.81), indicating that neutrophil-driven inflammatory programs are especially prominent during the transition to organ failure. This interpretation was supported by OLFM4, LTF, and CEACAM6, which were all upregulated in SAP vs MAP and co-selected by LASSO and random forest in ML-B.

OLFM4 is a neutrophil-associated granule protein expressed in a subset of neutrophils and has been linked to disease severity in systemic infectious and inflammatory states [14]. Lactoferrin (LTF) is a neutrophil-derived granule protein linked to NET-associated inflammatory responses [15]. CEACAM6 is expressed in neutrophils and may be associated with neutrophil activation-related functions [16]. The co-selection of OLFM4/LTF/CEACAM6 by LASSO and random forest, NETosis GSEA enrichment (NES=+1.98 for SAP vs MAP), and increasing neutrophil ssGSEA scores point to a neutrophil/NETosis axis associated with SAP progression. This is consistent with prior experimental work demonstrating that NETs promote trypsin activation, complement cascade engagement, and microvascular injury in murine AP models [5].

### 4.3. Concurrent activation of multiple inflammatory cell-death programs

Beyond NETosis, our GSEA analysis using custom gene sets revealed significant enrichment of inflammasome-, pyroptosis-, and ferroptosis-related signatures in both comparisons, whereas cuproptosis and necroptosis were not significantly enriched. This pattern is compatible with coordinated activation of several inflammatory cell death-related programs, while remaining selective rather than a non-specific increase across all curated cell-death signatures [6]. Pyroptosis has been increasingly implicated in AP, where inflammasome activation and gasdermin-mediated inflammatory cell death may contribute to pancreatic and extra-pancreatic injury amplification [17]. Recent studies have also implicated ferroptosis in AP pathophysiology, including oxidative stress amplification and intestinal barrier injury [18].

### 4.4. MRPL51 as a candidate marker associated with mitochondrial metabolic reprogramming

MRPL51, a mitochondrial ribosomal protein of the large subunit, was selected by all three ML-A algorithms and showed a high discovery-cohort raw-expression AUC (0.951) in SAP vs HC. This contrast compares severe-spectrum AP with healthy controls and is therefore less clinically stringent than distinguishing SAP from MAP. Its upregulation in SAP vs HC (log2FC=+1.09) is consistent with strong enrichment of oxidative phosphorylation (NES=+2.76), suggesting that MRPL51 reflects broad AP-associated oxidative-metabolic transcriptional changes rather than a severity-specific diagnostic marker. Mitochondrial dysfunction is increasingly recognized as a key component of AP pathophysiology, linking bioenergetic stress, autophagy failure, and inflammatory tissue injury [19]. MRPL51 specifically has not been extensively characterized in the AP context, and its role remains to be functionally validated.

### 4.5. C1QA links complement activation to macrophage-associated organ injury

C1QA, encoding the A chain of the complement C1q complex, was identified as an organ failure-specific candidate by the ML-B framework (log2FC=+1.38, AUC = 0.754). C1QA is predominantly secreted by monocytes and macrophages, and its upregulation is consistent with the progressive increase in M1 macrophage enrichment scores in ssGSEA. The complement system has been implicated in AP-associated systemic inflammation, with substantial crosstalk between complement activation and coagulation pathways contributing to microvascular injury and organ dysfunction [8]. C1q-related signaling has also been discussed in broader systemic inflammatory and critical illness contexts, supporting the broader relevance of complement-associated immune dysregulation beyond pancreatitis [20]. C1qa showed the most prominent macrophage-enriched expression pattern in the cross-species rat ileal scRNA-seq analysis. Its plasma detectability according to HPA annotation suggests potential accessibility as a circulating biomarker candidate, though clinical utility in AP requires prospective validation.

### 4.6. DACT1 downregulation suggests possible Wnt-related dysregulation during progression to organ failure

DACT1, a negative regulator of Wnt-related signaling, was specifically downregulated in SAP vs MAP (log2FC=−1.30, AUC = 0.758). DACT1 has been studied in developmental and cancer-related contexts as a regulator of Wnt pathway activity [21]. Wnt signaling has been implicated in pancreatic development, tissue homeostasis, and injury-repair responses, making Wnt-related dysregulation a biologically plausible axis for further investigation in AP [22]. Reduced DACT1 expression in the severe-spectrum group may suggest dysregulation of Wnt-related signaling during progression to organ failure. However, the role of DACT1 in AP has not been directly established, and this interpretation should be regarded as hypothesis-generating. Further mechanistic studies will be required to determine whether DACT1 has a functional role in AP-associated systemic inflammation or organ injury.

### 4.7. Stratified adaptive immune signature reduction

The stratified pattern of adaptive immune signature reduction was a reproducible feature across the ssGSEA, GSEA, and WGCNA analyses. CD8 + T-cell effector and NK-cell-associated signatures were already markedly reduced in MAP and did not differ significantly between SAP and MAP, indicating these reductions occur as a pan-AP phenomenon. CD4 + T-cell and Treg-associated signatures, however, showed additional reduction in SAP relative to MAP, indicating that this second tier of adaptive immune signature reduction is more closely associated with organ failure than with AP in general.

This pattern is consistent with the concept of CARS, in which early pro-inflammatory activation is accompanied or followed by systemic immune suppression [7]. The finding of reduced Treg-associated signatures in SAP vs MAP warrants particular discussion, as it appears to differ from prior work implicating activated regulatory T cells in severe AP, including studies suggesting that Treg activity may contribute to immunoregulatory and barrier-related responses during advanced disease [23]. One possible explanation is that bulk Treg-associated signatures may aggregate functionally distinct Treg states, as regulatory T-cell heterogeneity has been increasingly recognized in sepsis and other critical illness contexts [24]. This hypothesis cannot be resolved from bulk transcriptomic data alone and represents an important question for future flow cytometric and functional studies. The WGCNA brown module, negatively correlated with SAP severity (r=−0.607) and containing DACT1, CLEC4C, TRBV15, and KIR2DS4, provides network-level support for this adaptive immune suppression pattern.

### 4.8. Relationship to prior analyses of GSE194331 and existing AP transcriptomic studies

The dataset used in this study (GSE194331) was originally published by Nesvaderani et al., who identified novel pathways and potential biomarkers in SAP, focusing primarily on the SAP vs HC comparison [12]. The present study extends that work by introducing an explicit SAP vs MAP comparison, applying a triple-algorithm machine-learning strategy with intersection-based candidate selection, incorporating ssGSEA-based immune signature analysis and WGCNA, and using a rat single-cell dataset for cautious cell-type contextualization. Relative to pathway-focused bulk transcriptomic studies and more recent immune-cell-resolved analyses in SAP, multi-framework integrative designs that jointly combine pathway enrichment, immune signature scoring, and consensus machine learning within the same AP dataset remain relatively uncommon [12,13]. Our dual-framework design may provide a useful analytical template for other severity-stratified inflammatory disease datasets.

### 4.9. Study limitations

Several limitations should be acknowledged. First, all primary analyses were performed within a single discovery cohort (GSE194331), with a small severe-spectrum group (n = 30) and true SAP subgroup (n = 10). We searched for independent human peripheral-blood or PBMC RNA-seq cohorts of AP with severity labels suitable for sample-level external AUC validation, but no suitable dataset was identified beyond GSE194331. Therefore, the AUC values reported here should be interpreted as discovery-cohort estimates rather than externally validated diagnostic performance. Repeated stratified 5-fold cross-validation supported internal stability of the single-gene estimates, but prospective validation in independent cohorts remains necessary. Second, the MSAP and true SAP subgroups were combined to improve power for severe-spectrum analysis. Sensitivity analyses did not identify transcriptome-wide FDR-significant MSAP-vs-true-SAP differences, and selected adaptive immune ssGSEA signatures did not differ significantly; however, OLFM4 was higher in true SAP than in MSAP (FDR = 0.008), with LTF showing the same direction at nominal significance (p = 0.019; FDR = 0.057). This indicates that neutrophil/NETosis-associated signals may vary across severe-spectrum subgroups even though the combined group was used as a pragmatic power-preserving analytic choice. Third, the scRNA-seq contextualization dataset (GSE244963) was derived from rat ileal tissue rather than human peripheral blood; cross-species and cross-tissue differences must be considered when interpreting these results. Fourth, ssGSEA provides immune cell-associated signature enrichment scores rather than direct measurements of immune cell abundance or function; validation by flow cytometry in matched clinical samples would be required to confirm cellular interpretations. Fifth, the metadata available in GSE194331 were limited to group assignment information, without clinical severity scores (APACHE II, SOFA), detailed organ failure data, or longitudinal outcomes. Finally, protein-level and mechanistic validation were not performed, so the biological roles of MRPL51, C1QA, DACT1, and the NETosis triad remain hypothesis-generating.

## Conclusion

Integrative transcriptomic analysis of peripheral blood from acute pancreatitis patients identifies three discovery-level candidate genes (MRPL51, C1QA, DACT1) and a neutrophil-associated NETosis gene triad (OLFM4, LTF, CEACAM6) with potential relevance to progression from mild AP to the severe-spectrum AP group. A stratified adaptive immune signature reduction pattern, with CD8 + T-cell and NK-cell reductions as pan-AP phenomena and additional CD4 + T-cell and Treg-associated reductions as organ-failure-associated features, was supported by ssGSEA, GSEA, and WGCNA. Independent prospective validation and functional mechanistic studies are warranted to translate these findings toward clinical application.

### Use of AI-assisted language editing

During the preparation of this manuscript, the authors used ChatGPT (OpenAI) solely for English grammar correction and language polishing. All content was critically reviewed, revised, and approved by the authors, who take full responsibility for the final manuscript.

## Supporting information

S1 File Revised supplementary materials. This document contains Supplementary Methods SM 2.1–2.3, Supplementary Tables S1–S8, and the supplementary figure legends.
(DOCX)

S2 File Complete supplementary gene-set, ssGSEA-signature, and WGCNA data. This Excel workbook contains complete differential-expression gene lists, custom gene-set membership, ssGSEA signature definitions, per-sample ssGSEA scores, three-group ssGSEA summary statistics, WGCNA module summaries, complete module membership, and hub-gene data.
(XLSX)

S1 Fig Custom gene set GSEA results for inflammatory cell-death programs. Bubble plot showing enrichment of custom-curated gene sets in SAP vs HC and SAP vs MAP comparisons. Bubble color reflects the normalized enrichment score (NES), and bubble size reflects the absolute NES value. Non-significant gene sets are shown as grey circles. NES, normalized enrichment score; FDR, false discovery rate.
(TIFF)

S2 Fig scRNA-seq quality-control metrics for the rat ileal contextualization dataset. Violin plots show quality-control metrics across SAP (n = 3) and NC (n = 3) samples in GSE244963, including number of genes detected per cell, total UMI counts per cell, and percentage of mitochondrial reads. A total of 80,256 cells were retained after QC filtering (SAP n = 45,165; NC n = 35,091). NC, normal control; UMI, unique molecular identifier.
(TIFF)

S3 Fig WGCNA soft-thresholding power selection. Scale-free topology model fit (R2) and mean network connectivity are shown as functions of soft-thresholding power. The selected power beta = 9 (R2 = 0.806) balances scale-free topology fit and network connectivity in this small exploratory cohort.
(TIFF)

S4 Fig Expression of additional machine-learning candidate genes across HC, MAP, and SAP groups. Boxplots show log2-CPM expression levels of additional machine-learning candidate genes (OLFM4, IL10, KIR2DS4, LTF, ARG1, CLEC4C, CEACAM6, and TRBV15). OLFM4, LTF, and CEACAM6 are interpreted as a neutrophil-associated NETosis triad co-selected by LASSO and random forest in ML-B.
(TIFF)

S5 Fig Internal repeated cross-validation of core single-gene classifiers. Boxplots show repeated stratified 5-fold cross-validation AUC values across 20 repeats for MRPL51, C1QA, and DACT1. Triangles indicate apparent/raw-expression ROC values, diamonds indicate logistic-regression LOOCV estimates, and crosses indicate raw-expression reference AUC values. These analyses assess internal stability within the discovery cohort and do not constitute external validation.
(TIFF)
