# Supplementary material for: Integrative transcriptomic analysis of peripheral blood identifies NETosis-associated programs and a stratified adaptive immune suppression pattern in severe acute pancreatitis
Source: PLoS One. 2026 Sep 25;21(9):e0349692. doi: 10.1371/journal.pone.0349692 (PMC13614542; doi:10.1371/journal.pone.0349692)
Supplement: Supplementary file 1 [file pone.0349692.s001.docx]

**REVISED SUPPLEMENTARY MATERIALS**

Integrative Transcriptomic Analysis of Peripheral Blood Identifies NETosis-Associated Programs and a Stratified Adaptive Immune Suppression Pattern in Severe Acute Pancreatitis

**Supplementary Methods**

**SM 2.1 Custom Gene Set Construction**

Custom literature-derived gene sets were constructed to complement the curated MSigDB Hallmark, KEGG, and Reactome collections used in primary GSEA analyses. The additional gene sets covered inflammasome activation, pyroptosis, NETosis, PANoptosis, cuproptosis, necroptosis, ferroptosis, sepsis/organ failure, and mitochondrial dysfunction. Constituent genes were curated from pathway databases and published mechanistic literature, formatted as GMT-compatible gene sets, and analyzed together with the primary MSigDB collections. Complete gene membership is provided in Supplementary Table S6 and in S2 File.

**SM 2.2 scRNA-seq Custom Barcode Loader**

Raw count matrices from GSE244963 were provided in a non-standard 10x Genomics-like format in which barcode strings contained a numeric prefix separated by a space (e.g., "34 GTCGCTATA..."). Standard 10x loaders do not parse this format correctly. A custom Python loader was implemented in which barcode strings were processed using regular expression substitution to remove the numeric prefix and whitespace before AnnData object construction. This preprocessing step was implemented in step7_scrna_context.py and applied uniformly across all six sample directories (SAP-1, SAP-2, SAP-3, NC-1, NC-2, NC-3).

**SM 2.3 WGCNA Python Implementation**

Weighted gene co-expression network analysis was performed using the supplied Python script step6_wgcna.py, following the analytical workflow of the WGCNA R package. The top 5,000 most variable genes selected by median absolute deviation were used as input. Pairwise Pearson correlation matrices were transformed to an unsigned adjacency matrix using soft-thresholding power beta=9, selected as a compromise between scale-free topology fit (R2=0.806; Supplementary Figure S3) and network connectivity in this small exploratory cohort. Module eigengenes were correlated with an ordinal severity variable coded HC=0, MAP=1, and SAP=2.

**Supplementary Tables**

**Supplementary Table S1. GSEA pathway enrichment results (dual comparison).**

| **Pathway / Gene Set** | **Source** | **NES SAP vs HC** | **NES SAP vs MAP** | **FDR SAP vs HC** | **FDR SAP vs MAP** | **Mechanistic Axis** |
| --- | --- | --- | --- | --- | --- | --- |
| Oxidative Phosphorylation | Hallmark | +2.76 | +2.63 | <0.001 | <0.001 | Metabolic reprogramming |
| NETosis | KEGG | +2.34 | +1.98 | <0.001 | <0.001 | Innate immunity / NETosis |
| IL-6/JAK/STAT3 Signaling | Hallmark | +2.27 | — | <0.001 | ns | Inflammatory amplification |
| Complement & Coagulation | KEGG | +2.24 | +2.29 | <0.001 | <0.001 | Coagulation / Organ injury |
| Phagosome | KEGG | +2.23 | — | <0.001 | ns | Innate immunity |
| Lysosome | KEGG | +2.21 | — | <0.001 | ns | Innate immunity |
| Inflammatory Response | Hallmark | +2.08 | — | <0.001 | ns | Inflammatory amplification |
| Fc Gamma R-mediated Phagocytosis | KEGG | +2.05 | — | <0.001 | ns | Innate immunity |
| Reactive Oxygen Species | Hallmark | +2.01 | +2.20 | <0.001 | <0.001 | Oxidative stress |
| TNF-α / NF-κB Signaling | Hallmark | +1.99 | — | <0.001 | ns | Inflammatory amplification |
| mTORC1 Signaling | Hallmark | +1.95 | — | <0.001 | ns | Metabolic reprogramming |
| Coagulation | Hallmark | +1.90 | — | <0.001 | ns | Coagulation |
| Apoptosis | Hallmark | +1.88 | — | <0.001 | ns | PANoptosis |
| Glycolysis | Hallmark | +1.85 | +2.16 | <0.001 | <0.001 | Metabolic reprogramming |
| Ferroptosis | KEGG | +1.82 | +2.01 | <0.001 | <0.001 | PANoptosis |
| T Cell Receptor Signaling | KEGG | ns | <0 (suppressed) | ns | <0.05 | Adaptive immune suppression |
| INFLAMMASOME (custom) | Custom | +2.06 | +1.97 | <0.001 | <0.01 | PANoptosis |
| PYROPTOSIS (custom) | Custom | +1.99 | +1.64 | <0.001 | <0.05 | PANoptosis |
| NETosis (custom) | Custom | +1.81 | +1.98 | <0.01 | <0.01 | NETosis |
| PANoptosis (custom) | Custom | +1.74 | +1.55 | <0.01 | <0.05 | PANoptosis |
| FERROPTOSIS (custom) | Custom | +1.67 | +1.55 | <0.01 | <0.05 | PANoptosis |
| CUPROPTOSIS (custom) | Custom | +1.16 | +0.82 | ns | ns | — |
| NECROPTOSIS (custom) | Custom | +0.90 | −0.85 | ns | ns | — |

NES, normalized enrichment score. ns, not significant (FDR >= 0.05). Curated pathways were derived from MSigDB Hallmark, KEGG 2021 Human, and Reactome collections. Custom gene sets were constructed from published literature; complete membership is provided in Supplementary Table S6.

**Supplementary Table S2. ssGSEA immune signature statistics.**

| **Cell Type** | **Trend HC→SAP** | **FDR SAP vs HC** | **FDR SAP vs MAP** | **Immunological Interpretation** |
| --- | --- | --- | --- | --- |
| Neutrophils | ↑↑↑ (gradient) | <0.001 | <0.001 | Progressive innate activation |
| Monocytes | ↑↑↑ (gradient) | <0.001 | <0.001 | Progressive innate activation |
| Macrophages (M1) | ↑↑↑ (gradient) | <0.001 | <0.01 | Progressive innate activation |
| NK cells | ↓↓↓ (MAP→SAP ns) | <0.001 | ns | Pan-AP reduction (Tier 1) |
| CD8+ T effector | ↓↓↓ (MAP→SAP ns) | <0.001 | ns | Pan-AP reduction (Tier 1) |
| CD4+ T naive | ↓↓↓ (gradient) | <0.001 | <0.001 | Progressive reduction |
| CD4+ Treg | ↓↓ (SAP-specific) | <0.001 | <0.01 | Organ failure-associated (Tier 2) |
| pDC | ↓ (inferred) | — | — | Inferred from CLEC4C (ML-B) |

Significance was assessed by Kruskal-Wallis test with Dunn post-hoc testing and Benjamini-Hochberg FDR correction. Tier 1 indicates pan-AP immune reduction; Tier 2 indicates organ failure-associated additional reduction. Exact per-sample ssGSEA scores and three-group summary statistics are provided in the worksheets ssGSEA_scores_all_samples and ssGSEA_statistics_3groups, respectively, in S2 File.

**Supplementary Table S3. Machine-learning candidate genes with biological annotation.**

| **Gene** | **Framework** | **Selection evidence** | **log2FC** | **FDR** | **Discovery-cohort AUC** | **WGCNA module** | **HPA reliability** | **Biological annotation** |
| --- | --- | --- | --- | --- | --- | --- | --- | --- |
| **MRPL51** | **ML-A** | **LASSO+RF+SVM-RFE; n=3** | **+1.09** | **<0.001** | **0.951** | **-** | **Approved (intracellular)** | **Mitochondrial ribosomal protein; broad SAP-vs-HC oxidative/metabolic transcriptomic signal** |
| **C1QA** | **ML-B** | **LASSO+RF+SVM-RFE; n=3** | **+1.38** | **<0.001** | **0.754** | **Red (ID 6)** | **Supported (secreted)** | **Complement C1q A chain; macrophage-associated; plasma-detectable** |
| **DACT1** | **ML-B** | **LASSO+RF+SVM-RFE; n=3** | **-1.30** | **0.007** | **0.758** | **Brown (ID 3)** | **Enhanced (intracellular)** | **Wnt pathway regulator; reduced in SAP vs MAP** |
| OLFM4 | ML-B | LASSO+RF; n=2 | +2.26 | <0.001 | - | Red (ID 6) | - | Neutrophil-associated granule protein; NETosis triad member |
| IL10 | ML-B | LASSO+RF; n=2 | +1.88 | <0.001 | - | Red (ID 6) | - | Anti-inflammatory cytokine; CARS-related signal |
| LTF | ML-B | LASSO+RF; n=2 | +1.51 | 0.002 | - | Grey/unassigned | - | Neutrophil secondary granule protein; NETosis triad member |
| ARG1 | ML-B | LASSO+SVM-RFE; n=2 | +1.30 | 0.017 | - | Red (ID 6) | - | Myeloid immune suppression mediator |
| CEACAM6 | ML-B | LASSO+RF; n=2 | +1.10 | 0.034 | - | Grey/unassigned | - | Neutrophil activation marker; NETosis triad member |
| CLEC4C | ML-B | LASSO+RF; n=2 | -1.18 | 0.009 | - | Brown (ID 3) | - | pDC-associated marker; reduced in SAP vs MAP |
| TRBV15 | ML-B | LASSO+RF; n=2 | -1.03 | 0.008 | - | Brown (ID 3) | - | T-cell receptor component; consistent with adaptive immune suppression |
| KIR2DS4 | ML-A | LASSO+SVM-RFE; n=2 | -1.84 | 0.004 | - | Brown (ID 3) | - | NK-cell activating receptor; reduced in ML-A pairwise selection |

Core three-algorithm intersection candidates are MRPL51, C1QA, and DACT1. AUC values are discovery-cohort raw-expression ROC estimates matching Figure 4. OLFM4, LTF, and CEACAM6 form a neutrophil-associated NETosis triad but were co-selected by LASSO and random forest only (2 of 3 ML-B algorithms), not by all three algorithms. MRPL51 was excluded from WGCNA high-variance gene filtering because of low median absolute deviation.

**Supplementary Table S4. WGCNA co-expression module summary.**

| **Module** | **Numeric ID** | **r (SAP status)** | **r (Severity)** | **Direction** | **ML Candidate Genes** | **Biological Interpretation** |
| --- | --- | --- | --- | --- | --- | --- |
| Red | 6 | +0.428 | +0.623 | Positive | C1QA, OLFM4, IL10, ARG1 | Innate immune activation and tissue injury-associated |
| Brown | 3 | −0.481 | −0.607 | Negative | DACT1, CLEC4C, TRBV15, KIR2DS4 | Adaptive immune suppression-associated |
| Other modules | 0, 1, 2, 4, 5 | See S8 | See S8 | Lower magnitude or unassigned | LTF, CEACAM6 in grey/unassigned | Not highlighted as primary severity-associated modules; full module statistics and hub genes are provided in Supplementary Table S8. |

r, Pearson correlation coefficient between module eigengene and trait variable. Severity was coded as an ordinal variable (HC=0, MAP=1, SAP=2). Full module membership is provided in Supplementary Table S8 and in S2 File.

**Supplementary Table S5. Cross-species scRNA-seq contextualization of candidate gene expression.**

| **Gene (rat)** | **Cell cluster localization** | **Detection Status** | **Interpretation** |
| --- | --- | --- | --- |
| C1qa | Macrophage cluster | Detected | Most prominent macrophage-enriched expression; is consistent with macrophage origin of C1QA signal |
| Arg1 | Myeloid cluster | Detected | Consistent with myeloid immune suppression role |
| Ltf | Neutrophil-associated region | Detected | Is consistent with neutrophil origin of NETosis triad |
| Olfm4 | Scattered + partial epithelial | Detected | Consistent with dual identity: neutrophil activation and intestinal stem cell marker |
| Mrpl51 | Diffuse low expression | Detected | Consistent with housekeeping gene characteristics |
| Dact1 | Scattered distribution | Detected | Consistent with multi-tissue expression pattern |
| Il10 | Immune cell clusters | Detected | Present in immune cell populations |
| Clec4c | Not detected | Absent | May reflect low representation of pDC-like cells in intestinal tissue |

Dataset: GSE244963 (rat ileum; SAP n=3, NC n=3; 80,256 cells post-QC). These data provide supportive cross-species cell-type context and should not be interpreted as independent validation of human peripheral-blood biomarkers.

**Supplementary Table S6. Custom inflammatory cell-death and related pathway gene sets.**

| **Gene set** | **N genes** | **Genes** |
| --- | --- | --- |
| CUPROPTOSIS | 18 | FDX1, LIPT1, GLS, SLC31A1, PDHA1, NFE2L2, CDKN2A, PDHB, MT1X, DBT, DLAT, MTF1, LIAS, ATP7B, DLD, MT2A, MT1F, ATP7A |
| PYROPTOSIS | 16 | CASP3, CASP1, P2RX7, CASP5, NLRP3, CASP4, CXCL8, AIM2, PANX1, IL1B, HMGB1, GSDMD, NLRP1, CXCL1, PYCARD, IL18 |
| NECROPTOSIS | 19 | TNFRSF1A, CYLD, RIPK3, TNFAIP3, BIRC2, RIPK1, FADD, CASP8, TNFRSF10B, TRAF2, SHARPIN, PGAM5, TNFRSF10A, RNF31, RBCK1, TRAF5, TRADD, BIRC3, MLKL |
| PANoptosis_markers | 18 | TNFRSF1A, RIPK3, CASP3, CASP1, IL18, RIPK1, NLRP3, FADD, IL1B, APAF1, CASP8, HMGB1, GSDMD, CYCS, PARP1, PYCARD, ZBP1, MLKL |
| NETosis | 12 | MMP8, S100A9, PADI4, CXCL8, MMP9, S100A8, CEACAM8, MPO, CXCL1, S100A12, OLFM4, CD177 |
| INFLAMMASOME | 21 | RELA, CASP1, P2RX7, NLRP3, S100A9, CXCL8, S100A8, AIM2, TXNIP, PANX1, NFKB1, IL1B, HMGB1, NLRC4, GSDMD, NLRP1, RELB, NFKB2, TNF, PYCARD, IL18 |
| SEPSIS_organ_failure | 25 | PDCD1, RELA, IL10, STAT3, MMP8, S100A9, CXCL8, MMP9, S100A8, IL1RN, NFKB1, IL1B, HMGB1, TIGIT, MYD88, TLR4, MPO, CD274, CD14, FOXP3, CTLA4, ARG1, SOCS3, TNF, IL18 |
| MITOCHONDRIAL_DYSFUNCTION | 26 | HTRA2, ATG5, ULK1, MFN1, BNIP3, PARK7, BECN1, CASP9, BCL2, APAF1, BAX, OPA1, ENDOG, DIABLO, DNM1L, LRRK2, PGAM5, MFN2, SQSTM1, AIFM1, CYCS, ATG7, SNCA, FUNDC1, PINK1, TP53 |
| FERROPTOSIS_CURATED | 26 | SLC1A5, NFE2L2, LPCAT3, PHKG2, GLS2, ALOX5, TFRC, STEAP3, PTGS2, FTH1, ACSL3, TNFAIP3, HMOX1, DPP4, FANCD2, NFS1, CISD1, FTL, HSPB1, SLC40A1, SLC3A2, ACSL4, MGST1, SLC7A11, GPX4, SAT1 |

Gene sets are shown in GMT-compatible format. A long-format version is provided in S2 File.

**Supplementary Table S7. ssGSEA 10-gene immune signatures.**

| **Signature** | **N genes** | **Genes** |
| --- | --- | --- |
| Neutrophils | 10 | FCGR3B, CXCR1, CXCR2, CSF3R, S100A8, S100A9, MMP8, ELANE, MPO, CEACAM8 |
| Monocytes | 10 | CD14, FCGR1A, CSF1R, CCR2, S100A12, VCAN, THBS1, CD163, MRC1, ITGAM |
| Macrophages_M1 | 10 | CD80, CD86, IL1B, TNF, CXCL10, NOS2, STAT1, IRF5, SOCS3, IDO1 |
| NK_cells | 10 | NCAM1, KLRD1, GNLY, NKG7, PRF1, GZMB, KIR2DL1, KIR3DL1, FCGR3A, NCR1 |
| CD4_T_naive | 10 | CD4, CCR7, SELL, TCF7, LEF1, FOXP1, KLF2, IL7R, CD27, CD28 |
| CD4_Treg | 10 | FOXP3, CTLA4, TIGIT, IL2RA, IKZF2, ENTPD1, TNFRSF4, TNFRSF9, LAYN, BATF |
| CD8_T_effector | 10 | CD8A, CD8B, GZMK, GZMA, IFNG, TBX21, EOMES, PRF1, CCL4, CCL5 |
| CD8_T_exhausted | 10 | PDCD1, HAVCR2, LAG3, TIGIT, CTLA4, TOX, ENTPD1, CD244, CXCL13, BATF |
| Ferroptosis_def | 10 | GPX4, SLC7A11, NFE2L2, FTH1, HMOX1, SLC40A1, CISD1, ACSL3, MGST1, FTL |

Each immune signature contains 10 marker genes used for single-sample enrichment scoring.

**Supplementary Table S8. WGCNA module membership and hub-gene summary.**

| **Module** | **Module ID** | **N genes in module** | **r (SAP status)** | **r (Severity)** | **P (Severity)** | **ML candidate genes** | **Top hub genes** |
| --- | --- | --- | --- | --- | --- | --- | --- |
| Turquoise | 1 | 44 | -0.085 | -0.084 | 0.366 | (none) | OAS3, HERC5, RSAD2, CMPK2, IFI44L |
| Blue | 2 | 35 | -0.013 | -0.198 | 0.031 | (none) | RPL13AP25, RPL3P4, ENSG00000227615, RPLP0P9, RPL37P2 |
| Brown | 3 | 2309 | -0.481 | -0.607 | <0.001 | DACT1, CLEC4C, TRBV15, KIR2DS4 | ATP8B2, CD96, ITK, PLCG1, BCL11B |
| Yellow | 4 | 335 | 0.173 | 0.087 | 0.349 | (none) | GMPR, DMTN, BCL2L1, TAL1, YBX3 |
| Green | 5 | 43 | 0.179 | 0.267 | 0.003 | (none) | RPL23, RPS3A, RPL31, RPS7, RPL9 |
| Red | 6 | 2035 | 0.428 | 0.623 | <0.001 | C1QA, OLFM4, IL10, ARG1 | IFNGR2, AQP9, PYGL, RTN3, ALOX5 |
| Grey (unassigned) | 0 | 199 | - | - | - | LTF, CEACAM6 | (not applicable) |

The table summarizes module size, module-trait correlations, candidate-gene membership, and top hub genes. The complete 5,000-gene module membership table is provided in S2 File (sheet WGCNA_full_membership).

**Supplementary Figure Legends**

**Supplementary Figure S1. Custom gene set GSEA results for inflammatory cell death programs.**

Bubble plot showing enrichment of custom-curated gene sets in SAP vs HC and SAP vs MAP comparisons. Bubble color reflects the normalized enrichment score (NES), and bubble size reflects the absolute NES value. Non-significant gene sets are shown as grey circles. NES, normalized enrichment score; FDR, false discovery rate.

**Supplementary Figure S2. scRNA-seq quality control metrics for the rat ileal contextualization dataset.**

Violin plots show quality control metrics across SAP (n=3) and NC (n=3) samples in GSE244963, including number of genes detected per cell, total UMI counts per cell, and percentage of mitochondrial reads. A total of 80,256 cells were retained after QC filtering (SAP n=45,165; NC n=35,091). NC, normal control; UMI, unique molecular identifier.

**Supplementary Figure S3. WGCNA soft-thresholding power selection.**

Scale-free topology model fit (R2) and mean network connectivity are shown as functions of soft-thresholding power. The selected power beta=9 (R2=0.806) balances scale-free topology fit and network connectivity in this small exploratory cohort.

**Supplementary Figure S4. Expression of additional machine-learning candidate genes across HC, MAP, and SAP groups.**

Boxplots show log2-CPM expression levels of additional machine-learning candidate genes (OLFM4, IL10, KIR2DS4, LTF, ARG1, CLEC4C, CEACAM6, and TRBV15). OLFM4, LTF, and CEACAM6 are interpreted as a neutrophil-associated NETosis triad co-selected by LASSO and random forest in ML-B.

**Supplementary Figure S5. Internal repeated cross-validation of core single-gene classifiers.**

Boxplots show repeated stratified 5-fold cross-validation AUC values across 20 repeats for MRPL51, C1QA, and DACT1. Triangles indicate apparent/raw-expression ROC values, diamonds indicate logistic-regression LOOCV estimates, and crosses indicate raw-expression reference AUC values. These analyses assess internal stability within the discovery cohort and do not constitute external validation.
